# Pseudodementia or depression? An unresolved issue. Cognitive alterations in a population of geriatric patients

**DOI:** 10.1192/j.eurpsy.2023.782

**Published:** 2023-07-19

**Authors:** M. Violi, B. Buccianelli, M. Simoncini, L. Massoni, F. Pardini, L. Massa, S. Palermo, A. Arone, M. G. Carbone, D. Marazziti, L. Dell’Osso

**Affiliations:** DEPARTEMENT OF CLINICAL AND EXPERIMENTAL MEDICINE, UNIVERSITY OF PISA, PISA, Italy

## Abstract

**Introduction:**

The relationship between mood disorders, particularly depression and cognitive impairment is complex. The symptoms of depression in the elderly include confusion, sleep alterations, low concentration, cognitive deficits, and somatic complaints that may are also present in dementia, with depression being often a prodrome.

**Objectives:**

The present study aimed at investigating the presence of cognitive disturbances in outpatients over 65 years of age consulting us for a mood episode, as well as to investigate the possible relationships between cognitive and depressive symptoms.

**Methods:**

The study included 57 older patients attending the Psychiatric Clinic of Pisa, with a diagnosis of a major mood episode according to DSM-5 criteria. The psychometric scales included: Hamilton Depression Rating Scale (HAM-D), Beck Inventory Scale (BDI), Geriatric Depression Scale (GDS), to measure the severity of depression; Short Psychiatric Evaluation Schedule (SPES), to assess organic mental deficits; Cornell Scale for Depression in Dementia (CSDD), to assess depression in people with dementia*; Adult Autism Subthreshold (AdAS) Spectrum, to evaluates the eventual presence of specific features of the autistic spectrum disorder(ASD).* Moreover, patients were also assessed for cognitive screening with Montreal Cognitive Assessment (MoCA), Frontal Assessment Battery (FAB), Mini-Mental State Examination (MMSE).

**Results:**

The HAM-D total score was 10.18±6.33, that of BDI 12.79± 9.89, that of GDS 12.69±8.25 and that of CSDD 8.35±6.25. The showed a MoCA value was 21.30±4.86, that of FAB 14.12±3.92, and that of MMSE 25.06±4.20. The MoCA total score positively correlated with those of the FAB and of the MMSE, while the FAB score with the MMSE score. A positive correlation was found between SPES and the HAM-D, BDI, CSDD and GDS total scores. The AdAS score positively correlated with that of MMSE. By correlating scores of depressive dimensions with those of cognitive functions, a positive correlation was noted between FAB total score and those of the HAM-D, BDI, CSDD and SPES

**Conclusions:**

These findings suggest a possible link between the presence of ASD and depressive symptoms from the one side and cognitive performance and executive functions from the another side.

**Disclosure of Interest:**

None Declared

